# Dieulafoy’s Disease in Pregnancy: Pathophysiology, Clinical Presentation, and Management—A Case Report with Narrative Literature Review

**DOI:** 10.3390/jcm15051894

**Published:** 2026-03-02

**Authors:** Sophia Peretti, Elisabetta Dolfin, Silvana Ardunio, Luca Marozio, Maurizio Di Serio, Alberto Revelli

**Affiliations:** 1 SCDU1 Gynecology and Obstetrics, S. Anna Hospital, University of Torino, 10126 Torino, Italy; sophia.peretti@edu.unito.it (S.P.); luca.marozio@unito.it (L.M.); 2 Gynecology and Obstetrics, Cardinal Massaia Hospital, 14100 Asti, Italy; edolfin@asl.at.it (E.D.); mdiserio@asl.at.it (M.D.S.); 3 SCDU2 Gynecology and Obstetrics, S. Anna Hospital, University of Torino, 10126 Torino, Italy; arduino.silvana70@gmail.com

**Keywords:** Dieulafoy’s disease, gastrointestinal bleeding, anemia in pregnancy, endoscopy in pregnancy, vascular lesions, assisted reproductive technology

## Abstract

**Background:** Dieulafoy’s disease is a rare vascular anomaly of the gastrointestinal tract and an uncommon cause of acute upper gastrointestinal bleeding. Its occurrence during pregnancy is exceptionally rare, and the available literature is limited to isolated case reports that almost invariably describe acute and overt hemorrhagic presentations. As a result, atypical or clinically silent forms of the disease during pregnancy remain poorly characterized. **Objective:** To report an atypical case of Dieulafoy’s disease during pregnancy, presenting exclusively with severe progressive anemia in the absence of gastrointestinal symptoms and to contextualize this observation through a focused narrative review of the literature. **Methods:** An illustrative clinical case is presented, followed by a narrative review of the available literature on Dieulafoy’s disease in pregnancy. Particular attention was given to pregnancy-related physiological and hormonal adaptations, diagnostic challenges, therapeutic strategies, and reported maternal–fetal outcomes. All published cases identified in the literature were reviewed and summarized. **Results:** In the general population, Dieulafoy’s disease typically presents with sudden and overt gastrointestinal bleeding and is most commonly localized in the proximal stomach. In pregnancy, reported cases are rare and have almost exclusively involved acute hemorrhage occurring in the second or third trimester, frequently requiring urgent endoscopic intervention. Mechanical endoscopic hemostasis represents the treatment of choice and is generally associated with favorable maternal and fetal outcomes. In contrast, the illustrative case described herein demonstrates a clinically silent presentation, characterized by severe and progressive anemia without hematemesis, melena, or hematochezia, resulting in delayed diagnosis until upper gastrointestinal endoscopy identified multiple actively bleeding gastric Dieulafoy’s lesions. **Conclusions:** Dieulafoy’s disease should be considered in the differential diagnosis of severe, unexplained, or transfusion-dependent anemia during pregnancy, even in the absence of overt gastrointestinal bleeding. Pregnancy-related physiological adaptations may mask classic symptoms and complicate timely diagnosis. When clinically indicated, upper gastrointestinal endoscopy is safe and effective during pregnancy and remains central to both diagnosis and management. Increased awareness of atypical presentations may facilitate earlier recognition and improve maternal and fetal outcomes.

## 1. Introduction

Dieulafoy’s disease is a rare, potentially life-threatening vascular anomaly of the gastrointestinal tract, accounting for approximately 1–2% of acute upper gastrointestinal bleeding in the general population [1,2,3]. First described by Georges Dieulafoy in 1898, it is characterized by the presence of a large-caliber submucosal artery that fails to undergo physiological tapering as it approaches the mucosal surface [1]. Any minimal erosion of the overlying mucosa may expose the vessel and result in sudden arterial hemorrhage in the absence of peptic ulceration, inflammation, or malignancy. In non-pregnant patients, Dieulafoy’s disease typically presents with overt gastrointestinal bleeding, including hematemesis, melena, or hematochezia and may lead to hemodynamic instability or hemorrhagic shock [2,3]. Upper gastrointestinal endoscopy represents the diagnostic modality of choice, allowing both definitive diagnosis and immediate therapeutic intervention. Endoscopic mechanical hemostasis, such as hemoclip placement or band ligation, is widely regarded as first-line therapy, with high success rates and low recurrence risk [2,3,4].

The occurrence of gastrointestinal Dieulafoy’s disease during pregnancy is exceedingly rare. To date, the available literature is limited to a very small number of isolated case reports and small case series, reflecting both the exceptional rarity of the condition and a publication bias toward acute and dramatic hemorrhagic presentations [5,6,7]. Notably, nearly all cases reported in pregnant patients have been characterized by sudden and clinically evident gastrointestinal bleeding requiring urgent intervention [5,6,7], leaving the full clinical spectrum of disease expression during pregnancy incompletely defined. However, pregnancy may imply variations in the clinical picture as well as unique diagnostic and therapeutic challenges related to physiological hemodynamic adaptations, hormonally mediated vascular changes, fetal safety considerations, as well as the need for multidisciplinary management [8,9,10,11,12,13,14,15,16,17,18,19,20]. A substantial body of literature describes the anatomical features, pathophysiology, and endoscopic management of Dieulafoy’s lesions in the general population [1,2,3,4,21,22], as well as the systemic hemodynamic and vascular adaptations that occur during pregnancy [8,9,10,11,12,13,14,15,16,17,18,19,20]. Considering these physiological modifications may provide a useful interpretative framework for understanding atypical clinical presentations in pregnant patients. In this context, we report an illustrative case of Dieulafoy’s disease during pregnancy characterized by a clinically silent course, presenting exclusively with severe and progressive anemia in the absence of overt gastrointestinal symptoms. This unusual presentation prompted a focused narrative review of the literature aimed at contextualizing the case within current knowledge and exploring potential pregnancy-specific mechanisms underlying atypical disease expression. Accordingly, the present article is structured as a case-driven narrative review. The illustrative case is presented first, followed by a comprehensive synthesis of available evidence on the pathophysiology, clinical presentation, diagnostic approach, and management of gastrointestinal Dieulafoy’s disease in pregnancy. Indeed, all published cases of gastrointestinal Dieulafoy’s disease in pregnancy were systematically reviewed herein, and their main clinical features, management strategies, and maternal–fetal outcomes are summarized in Table S1. By integrating a rare and atypical clinical observation with a structured review of existing evidence, this work aims at broadening the currently recognized clinical spectrum of gastrointestinal Dieulafoy’s disease in pregnancy, highlighting relevant diagnostic pitfalls, and reinforcing the importance of considering gastrointestinal causes in pregnant patients with unexplained anemia.

## 2. Epidemiology, Anatomy, Clinical Picture, Diagnosis and Treatment of Dieulafoy’s Disease

Dieulafoy’s disease is an uncommon cause of gastrointestinal bleeding, accounting for approximately 1–2% of cases of acute upper gastrointestinal hemorrhage in the general population [2,3]. It is most frequently reported in adults, and has been described with a male predominance, although the mechanisms underlying sex-related differences remain poorly understood [2].

Dieulafoy’s lesions predominantly involve the stomach, which accounts for approximately 70–75% of reported cases, with a characteristic localization along the lesser curvature of the proximal stomach near the gastroesophageal junction [2,3,4]. Extra-gastric involvement has been documented in up to 25–30% of cases and includes the duodenum, small intestine, colon, rectum, and, more rarely, the esophagus [2,3,4]. Lesions at extra-gastric sites may be underdiagnosed due to their intermittent bleeding pattern and the technical challenges associated with endoscopic visualization of distal segments of the gastrointestinal tract [2,3]. In pregnancy, epidemiological data are limited to isolated case reports and small case series, precluding reliable estimates of incidence or prevalence. Nevertheless, available reports consistently indicate that gastric localization remains the most frequent site of involvement, mirroring the anatomical distribution observed in non-pregnant population [2,3,4,5,6,7].

Dieulafoy’s disease most commonly presents as sudden, intermittent gastrointestinal bleeding that may be massive, recurrent, and potentially life-threatening; it more often occurs in patients without prodromal symptoms or pre-existing gastrointestinal disease [1,2,3]. The bleeding is arterial in origin, and characteristically appears disproportionate to the size of the mucosal defect, which is typically minute and barely visible at endoscopic examination [2,3]. As a result, the severity of hemorrhage is often unexpected and may not correlate with endoscopic findings, contributing to diagnostic difficulty, particularly in the absence of active bleeding at the time of evaluation [3,4]. The clinical picture is often dominated by overt bleeding manifestations such as hematemesis or melena, and may rapidly progress to hemodynamic instability or hemorrhagic shock [2,3]. However, a defining feature of Dieulafoy’s disease is its intermittent bleeding pattern, which may result in spontaneous temporary hemostasis and lead to delayed or missed diagnosis, particularly when bleeding has ceased at the time of evaluation [3,4]. In some patients, repeated subacute or occult bleeding episodes may manifest as chronic iron-deficiency anemia rather than acute hemorrhage, further complicating clinical recognition and emphasizing the need for a high index of suspicion [3].

Upper gastrointestinal endoscopy is considered the diagnostic modality of choice and plays a central role in both the identification and immediate management of Dieulafoy’s lesions [2,3,4]. Characteristic endoscopic diagnostic criteria include active arterial spurting or micropulsatile bleeding from a minute mucosal defect, visualization of a protruding submucosal vessel with or without active bleeding, or the presence of a densely adherent clot attached to otherwise normal-appearing mucosa, in the absence of associated ulceration or mass lesions (Table S2) [2,3,4]. Diagnosis may be technically challenging due to the small size of the lesion, lack of surrounding inflammatory changes, and frequent absence of active bleeding during endoscopy. Consequently, initial endoscopic evaluation may be non-diagnostic in a subset of patients, and repeated endoscopy is often required when clinical suspicion persists [3,4,22]. When standard endoscopic evaluation fails to identify the bleeding source or when bleeding recurs despite initial therapy, adjunctive diagnostic modalities may be considered. Angiography or computed tomography angiography may demonstrate active contrast extravasation during ongoing hemorrhage, and can offer a therapeutic option via selective embolization in refractory cases [3,4,22]. Capsule endoscopy and deep enteroscopy may be useful in selected patients with obscure gastrointestinal bleeding when a small-bowel source is suspected, although their role is limited in the setting of acute, hemodynamically significant bleeding [3,4,22]. Finally, Dieulafoy’s disease should be distinguished from other causes of upper gastrointestinal bleeding on the basis of clinical context and endoscopic findings, including peptic ulcer disease, esophageal varices, Mallory–Weiss tears, and, rarely, gastric malignancy (Table S3) [2,3,22].

Once identified, endoscopic therapy represents the cornerstone of management for Dieulafoy’s disease. Mechanical hemostatic techniques, including hemoclip placement and band ligation, are widely regarded as first-line treatments and are associated with high rates of immediate hemostasis and low recurrence risk (Table S4) [2,3,4,21]. Injection therapies and thermal coagulation may be employed as adjunctive measures, but are generally considered less effective when used alone [3,4,21]. Advances in endoscopic recognition and treatment have markedly reduced the need for surgical intervention and significantly improved outcomes in non-pregnant populations [2,3,4,22].

## 3. Illustrative Case Report

A 37-year-old primigravida was referred at 18 weeks and 4 days gestational age for severe anemia (hemoglobin 6.6 g/dL). The patient’s medical history included Hashimoto’s thyroiditis on levothyroxine replacement therapy, a peri-membranous ventricular septal defect with a minimal and hemodynamically insignificant shunt, allergic asthma, migraine with aura, heterozygous methylenetetrahydrofolate reductase (MTHFR) mutation, and prior hysteroscopic metroplasty for a septate uterus. Pregnancy had been achieved using homologous in vitro fertilization (IVF), following a day-3 fresh embryo transfer. During the first trimester, the patient experienced a threatened miscarriage at 9 weeks of gestation, with heavy vaginal bleeding that spontaneously resolved. Prenatal ultrasound screening revealed fetal vertebral and renal anomalies suggestive of VACTERL association (Vertebral defects, Anal atresia, Cardiac defects, Tracheo-Esophageal fistula, Renal anomalies, and Limb abnormalities); the patient declined invasive prenatal diagnostic testing, and clinical follow-up was started, leading to a postnatal diagnosis of type II caudal regression syndrome. During pregnancy, this severely anemic patient required five hospital admissions and received a total of eleven units of packed red blood cells, as well as three infusions of ferric carboxymaltose. Despite extensive hematologic and systemic evaluations, the cause of anemia was not identified. Notably, the patient never reported hematemesis, melena, or hematochezia. A first esophagogastroduodenoscopy (EGDS) revealed two actively bleeding gastric vascular lesions consistent with Dieulafoy’s disease, located at the cardia and along the anterior wall of the gastric body (Images S1 and S2). Both lesions were characterized by arterial bleeding arising from minute mucosal defects in the absence of surrounding ulceration and were treated with endoscopic placement of two hemoclips. Four days later, a further episode of acute hemoglobin decline necessitated repeat upper gastrointestinal endoscopy: this second EGDS identified an additional actively bleeding gastric Dieulafoy’s lesion (Image S3), which was successfully treated with placement of a metallic hemoclip. Following endoscopic treatment, hemoglobin levels progressively improved, rising to 8.3 g/dL approximately two weeks after treatment and stabilizing at 10.6 g/dL one month later. Pregnancy progressed to term, and delivery occurred at 39 weeks and 2 days via vacuum-assisted vaginal delivery for non-reassuring cardiotocographic findings. Pre-delivery hemoglobin was 10.9 g/dL, and no postpartum anemia was observed.

## 4. Dieulafoy’s Disease in Pregnancy

Dieulafoy’s disease during pregnancy represents an exceptionally rare clinical entity, with the available literature limited to isolated case reports and very small case series [5,6,7]. Pregnancy-associated cases have been reported only sporadically, precluding the development of prospective studies and pregnancy-specific diagnostic or therapeutic guidelines.

All published cases of gastrointestinal Dieulafoy’s disease during pregnancy are summarized in Table S1, which provides a comparative overview of gestational age at presentation, clinical manifestations, lesion localization, diagnostic modality, therapeutic approach, and maternal–fetal outcomes. As shown in the table, reported cases have occurred predominantly during the second and third trimesters, and have almost invariably presented with clinically evident gastrointestinal bleeding (most commonly hematemesis and/or melena, and, less frequently, hematochezia or rectal bleeding), often accompanied by acute anemia and hemodynamic instability requiring urgent intervention [5,6,7]. With regard to anatomical distribution, Table S1 illustrates that pregnancy-associated cases have consistently involved gastric Dieulafoy’s lesions, most frequently located in the proximal stomach, along the lesser curvature or duodenum (third portion), mirroring the anatomical patterns observed in the general population [2,3,4,5,6,7]. 

Across previously published (Table S1), upper gastrointestinal endoscopy consistently represented the diagnostic modality of choice, allowing direct visualization of the lesion and simultaneous therapeutic intervention [5,6]. Diagnosis was uniformly triggered by overt hemorrhagic manifestations rather than by isolated laboratory abnormalities, reflecting a diagnostic pathway analogous to that observed in non-pregnant populations [5,6,7].

Notably, all published cases of Dieulafoy’s disease during pregnancy have been characterized by clinically evident hemorrhagic manifestations serving as the trigger for diagnostic evaluation and subsequent endoscopic confirmation. In contrast, our illustrative case described herein represents, to our knowledge, the first description of Dieulafoy’s disease in pregnancy presenting exclusively with severe and progressive anemia in the absence of overt gastrointestinal bleeding. This atypical clinical course expands the currently recognized disease spectrum and suggests that existing literature predominantly reflects symptomatic and acute presentations, while less typical or oligosymptomatic disease courses may be under-represented.

### 4.1. Pregnancy-Specific Modifications in the Pathophysiology of Dieulafoy’s Disease

Gastrointestinal Dieulafoy’s disease is characterized by the presence of a “caliber-persistent” submucosal artery, typically measuring 1–3 mm in diameter, that fails to undergo the physiological branching and tapering process as it approaches the mucosal surface [2,3]. As a consequence, a large-caliber arterial vessel runs immediately beneath the mucosa, rendering it particularly vulnerable to exposure and rupture following even minimal mucosal injury. Owing to the arterial nature of the lesion, bleeding is often sudden, brisk, and disproportionate to the size of the mucosal defect.

Although the precise pathogenesis of Dieulafoy’s disease remains incompletely elucidated, the prevailing hypothesis suggests a congenital or developmental abnormality in vascular remodeling, resulting in the persistence of a submucosal artery that would normally regress during embryologic development [2,3]. Over time, repetitive mechanical pulsation of this non-tapering vessel against the overlying mucosa may induce focal ischemia, progressive mucosal thinning, and eventually erosion. Importantly, this process occurs in the absence of surrounding inflammation, ulceration, or neoplastic change, thereby distinguishing Dieulafoy’s lesions from peptic ulcer disease and other more common causes of gastrointestinal bleeding [2,3,4]. Hemodynamic factors play a central role in determining both the timing and severity of bleeding episodes. Increased intraluminal arterial pressure within the persistent vessel, combined with local mucosal fragility, predisposes to sudden rupture and massive hemorrhage [2,4]. Additional local factors, including gastric acid exposure, peristaltic activity, and minor mechanical trauma, may further contribute to mucosal disruption and arterial exposure [2,3].

Pregnancy introduces a series of physiological adaptations that may significantly influence the clinical expression of a pre-existing Dieulafoy’s lesion, particularly during the second and third trimesters. Maternal plasma volume progressively increases throughout gestation, reaching approximately 40–50% above baseline, while cardiac output and heart rate rise in parallel, resulting in a substantial increase in systemic and regional blood flow [15,19,20]. These cardiovascular adaptations are essential to meet fetal and placental metabolic demand, but are also associated with increased intravascular pressure and shear stress within the maternal circulation [12,15]. Splanchnic and visceral circulations are similarly affected. The pregnancy-related increase in blood volume and cardiac output are accompanied by augmented perfusion of abdominal organs, potentially exposing pre-existing vascular anomalies of the gastrointestinal tract to higher flow states and mechanical stress [19,20,21]. In the presence of a caliber-persistent submucosal artery, such as the one characterizing Dieulafoy’s disease, these hemodynamic conditions may lower the threshold for vessel rupture or promote progressive mucosal erosion through repetitive arterial pulsation. Notably, these circulatory adaptations intensify as gestation advances and reach their peak during the late second and third trimesters, explaining the gestational age at presentation reported in the majority of published cases of Dieulafoy’s disease during pregnancy [5,6,7].

In addition to systemic hemodynamic changes, pregnancy is characterized by profound hormonally mediated vascular adaptations. Endogenous estrogen levels increase progressively throughout gestation and play a central role in physiological cardiovascular and vascular remodeling. Experimental and clinical studies have demonstrated that estrogens modulate endothelial function, promote nitric oxide–mediated vasodilation, enhance vascular endothelial growth factor (VEGF) expression, and contribute to angiogenesis and structural remodeling of the vascular wall [9,10,11,15,16]. These mechanisms are essential for normal maternal cardiovascular adaptation, but are also associated with increased vascular permeability, augmented mucosal vascularity, and enhanced vascular fragility [8,10,13,14,15,16,20]. Estrogen-driven vascular remodeling occurs in parallel with the aforementioned hemodynamic adaptations, including plasma volume expansion and redistribution of regional blood flow [8,10,13,15,16,17,18]. These processes become particularly pronounced during the second and third trimesters, when circulating estrogen levels peak and endothelial nitric oxide–dependent pathways are most active [8,9,10,13,17]. In this context, a pre-existing caliber-persistent submucosal artery may be exposed to increased intravascular pressure and mucosal stress, potentially lowering the threshold for erosion of the overlying mucosa and arterial exposure. Although no studies have demonstrated a causal association between estrogen exposure and the development of Dieulafoy’s lesions, pregnancy-related hormonal and hemodynamic adaptations may plausibly affect the clinical expression of a pre-existing vascular anomaly. This interpretation is supported by the observation that the vast majority of reported cases of Dieulafoy’s disease during pregnancy appear in the second or third trimester, coinciding with the period of maximal endogenous estrogen exposure and cardiovascular adaptation [5,6,7].

### 4.2. Clinical Presentation in Pregnancy

In contrast to the heterogeneous clinical spectrum observed outside pregnancy, reported cases in pregnant patients display a striking clinical uniformity. Nearly all published reports describe symptomatic presentations characterized by massive or recurrent upper gastrointestinal bleeding—manifesting as hematemesis, melena, hematochezia, or rectal bleeding—and frequently associated with hemodynamic compromise requiring urgent endoscopic hemostasis [5,6,7]. In these cases, anemia has consistently occurred in conjunction with clinically apparent hemorrhage, in keeping with classical descriptions of Dieulafoy’s disease in non-pregnant populations [2,3,4]. The clinical course observed in the present illustrative case substantially broadens this spectrum: the patient presented exclusively with severe and progressive anemia in the complete absence of gastrointestinal symptoms, and diagnosis was delayed because anemia was initially attributed to more common obstetric and hematologic causes. Some physiological adaptations of pregnancy—including progressive plasma volume expansion, hemodilution, increased cardiac output, and redistribution of regional blood flow—may allow substantial blood loss to occur before classical hemorrhagic symptoms become clinically apparent [8,13,17,18,19,20]. As a consequence, atypical bleeding patterns or clinically silent disease courses may be masked during pregnancy, contributing to delayed recognition and underscoring the importance of maintaining diagnostic vigilance in cases of unexplained, progressive, or transfusion-dependent anemia.

### 4.3. Maternal and Fetal Outcomes

When promptly recognized and appropriately managed, maternal and fetal outcomes in the reported cases of Dieulafoy’s disease during pregnancy are generally favorable. Across published cases, maternal morbidity has been primarily related to the severity and duration of hemorrhage at presentation; acute maternal complications included significant anemia requiring blood transfusion and, in some instances, hemodynamic instability necessitating urgent intervention [5,6,7]. Once effective endoscopic hemostasis was achieved, maternal clinical course was typically uncomplicated, with stabilization of hemoglobin levels and no reported recurrence of bleeding during the remainder of pregnancy [5,6,7]. Importantly, no maternal complications directly attributable to upper gastrointestinal endoscopy were reported in pregnancy-associated cases of Dieulafoy’s disease [23,24]. This observation is consistent with broader evidence supporting the safety of esophagogastroduodenoscopy during pregnancy when performed according to established guidelines [25].

Fetal outcomes reported in association with Dieulafoy’s disease during pregnancy have likewise been predominantly favorable. Available evidence indicates that fetal prognosis is largely determined by the rapid restoration of maternal hemodynamic stability and correction of anemia, rather than by the vascular lesion itself [5,6,7]. In cases where maternal bleeding was promptly controlled, pregnancies generally progressed to term without adverse fetal sequelae. Adverse fetal outcomes, including preterm delivery or fetal distress, have been reported primarily in the context of massive maternal hemorrhage, prolonged hemodynamic instability, or delayed intervention [5,6,7]. These events appear to be secondary to maternal hypovolemia and compromised uteroplacental perfusion. When appropriate precautions were observed, no fetal complications attributable to endoscopic procedures were reported [23,24]. In the present illustrative case, timely endoscopic clip placement resulted in immediate control of bleeding and progressive correction of maternal anemia, allowing pregnancy to continue safely to term with a favorable fetal outcome.

Overall, current evidence supports the conclusion that Dieulafoy’s disease during pregnancy, although potentially life-threatening at presentation, is associated with good maternal and fetal outcomes when promptly diagnosed and managed with endoscopic techniques. Diagnostic delay, particularly in atypical presentations lacking overt gastrointestinal bleeding, remains the primary factor contributing to adverse outcomes, underscoring the importance of early consideration of gastrointestinal etiologies in pregnant patients with severe or unexplained anemia.

### 4.4. Diagnostic Approach in Pregnant Patients

While in the general population diagnostic pathways are usually triggered by overt gastrointestinal hemorrhage, pregnancy introduces additional complexity, as physiological changes and obstetric considerations may obscure or delay recognition of gastrointestinal etiologies [2,3,4,5,6,7]. Importantly, the diagnostic criteria for Dieulafoy’s disease do not differ between pregnant and non-pregnant patients. Endoscopic diagnosis relies, in fact, on the same well-established criteria described in the general population, including: (i) active arterial spurting or micropulsatile bleeding from a minute mucosal defect; (ii) visualization of a protruding submucosal vessel through otherwise normal-appearing mucosa, with or without active bleeding; or (iii) a fresh, densely adherent clot with a narrow point of attachment to normal mucosa in the absence of ulceration or a mass lesion (Table S2) [2,3,4]. These criteria are particularly relevant in pregnancy, as the lesion is often extremely small and bleeding may be intermittent, increasing the likelihood of an initially non-diagnostic examination [3,4].

Non-invasive diagnostic tools, such as fecal occult blood testing, have limited sensitivity in the setting of Dieulafoy’s disease due to its intermittent bleeding pattern, and may yield false-negative results [2,3,4]. Therefore, a single negative test should not preclude further investigation when clinical suspicion remains high. Upper gastrointestinal endoscopy remains the diagnostic gold standard for Dieulafoy’s disease during pregnancy, as it allows direct visualization of the lesion and immediate therapeutic intervention [1,2,3,4]. The safety of esophagogastroduodenoscopy in pregnancy has been evaluated in multiple observational studies and expert consensus guidelines, which consistently support its use when clinically indicated. Multicenter experiences have demonstrated no increased risk of fetal malformations, pregnancy loss, or preterm labor attributable to endoscopy itself [5,6,7,24]. Across all published cases of Dieulafoy’s disease in pregnancy, endoscopy played a central diagnostic and therapeutic role, without reported procedure-related fetal complications [5,6,7]. To minimize maternal and fetal risk, current recommendations include left lateral maternal positioning to avoid aortocaval compression, judicious use of sedative agents with established safety profiles, avoidance of excessive gastric insufflation, and continuous maternal monitoring, with fetal heart rate monitoring when gestational age permits [24,25]. Conscious sedation is generally preferred, while deeper levels of sedation or general anesthesia should be reserved for selected cases following multidisciplinary discussion. Repeated endoscopy should be considered in patients with persistent or transfusion-dependent anemia, recurrent hemoglobin decline, or ongoing clinical concern despite an initially non-diagnostic examination [3,4,22].

Adjunctive diagnostic modalities may be considered in selected cases. Angiography or computed tomography angiography can identify active contrast extravasation during ongoing hemorrhage and may provide a therapeutic option through selective embolization; however, their use in pregnancy is constrained by concerns regarding fetal radiation exposure, and should be reserved for life-threatening situations after multidisciplinary evaluation [3,4,22]. Capsule endoscopy and deep enteroscopy may be useful for obscure gastrointestinal bleeding outside pregnancy, but their role during gestation is limited by the lack of therapeutic capability and insufficient safety data [3,25].

The differential diagnosis of gastrointestinal bleeding and anemia in pregnancy mirrors that of the general population, encompassing peptic ulcer disease, esophageal varices, Mallory–Weiss tears, and, more rarely, gastric malignancy (Table S3) [2,3,22]. What differs in pregnancy is not the diagnostic framework itself, but rather the clinical context, in which physiological hemodilution, iron deficiency, or obstetric bleeding are frequently presumed causes of anemia, potentially delaying gastrointestinal investigation.

A stepwise diagnostic approach for suspected Dieulafoy’s disease in pregnancy is proposed and summarized in Figure S1.

### 4.5. Therapeutic Strategies in Pregnancy

The management of Dieulafoy’s disease during pregnancy is primarily directed toward achieving rapid and durable hemostasis while minimizing maternal and fetal risk. Given the exceptional rarity of this condition in pregnant patients, no pregnancy-specific therapeutic guidelines exist, and current management is largely extrapolated from evidence in the non-pregnant population and from isolated case reports and small case series in pregnancy [5,6,7]. A stepwise, multidisciplinary approach to treatment is summarized in Figure S1.

Endoscopic therapy represents the cornerstone of treatment for Dieulafoy’s disease and is considered the first-line therapeutic approach in both pregnant and non-pregnant patients [1,2,3,4]. Its central role derives from the ability to achieve rapid diagnosis and immediate hemostasis in a single procedure, a feature that is particularly relevant in pregnancy, where ongoing bleeding or progressive anemia may pose substantial risks to maternal and fetal well-being. Mechanical endoscopic techniques, such as hemoclip placement and endoscopic band ligation, are generally preferred because they provide effective and durable hemostasis with low rebleeding rates and a favorable safety profile [2,3,4,21]. In the limited number of pregnancy-associated cases reported to date, endoscopic hemostasis has been consistently successful, even when performed emergently in the setting of massive upper gastrointestinal bleeding [5,6,7]. Notably, these interventions have not been associated with procedure-related fetal complications, further supporting their use when clinically indicated. In the case presented herein, endoscopic clip placement resulted in prompt and definitive control of bleeding despite the absence of overt gastrointestinal hemorrhage.

Injection therapy with diluted epinephrine may be employed as an adjunct to mechanical methods to improve visualization and facilitate initial bleeding control; however, it is not recommended as monotherapy because of higher rates of rebleeding [3]. Thermal coagulation techniques, although effective in the general population, should be used with caution during pregnancy, as pregnancy-related vascular changes and altered tissue perfusion may theoretically increase the risk of deep tissue injury or perforation [2,3,4]. Consequently, when endoscopic treatment is required during gestation, mechanical methods are generally favored whenever feasible.

As emphasized in Figure S1, supportive management plays a crucial role in the overall treatment strategy and includes red blood cell transfusion, iron supplementation, and close monitoring of maternal hemoglobin levels. In cases of severe anemia or hemodynamic compromise, prompt transfusion should not be delayed, as maternal hypovolemia and hypoxia pose significant risks to uteroplacental perfusion and fetal oxygenation and both maternal and fetal well-being [5,6,7]. From an obstetric perspective, management should prioritize optimization of uteroplacental perfusion and avoidance of iatrogenic preterm delivery whenever possible. Available reports indicate that, once maternal hemodynamic stability is restored following successful endoscopic hemostasis, pregnancy can often safely continue to term, with favorable neonatal outcomes [5,6,7].

As outlined in the proposed diagnostic and therapeutic flowchart (Figure S1), angiographic embolization may be considered as a second-line therapeutic option in cases of persistent or recurrent bleeding when endoscopic therapy fails, is technically unsuccessful, or cannot be promptly performed [3,4,22].

In pregnant patients, the role of angiography is inherently limited by concerns regarding fetal radiation exposure. Although modern angiographic techniques can significantly reduce radiation dose through shielding, collimation, and minimization of fluoroscopy time, angiography should be reserved for life-threatening hemorrhages, in which ongoing bleeding poses an immediate risk to maternal survival and endoscopic options have been exhausted [25]. When angiographic intervention is deemed unavoidable, a multidisciplinary approach involving interventional radiologists, obstetricians, anesthesiologists, and neonatologists is essential to balance maternal benefit against potential fetal risk, and to implement all available strategies to minimize radiation exposure.

Surgical intervention represents a last-resort option for refractory Dieulafoy’s disease when both endoscopic and angiographic approaches are unsuccessful or unavailable. Historically, surgery was frequently required prior to advances in endoscopic therapy; however, it is now rarely indicated and is associated with substantially higher maternal and fetal morbidity, particularly when performed emergently during pregnancy [2,3,4,22]. Surgical procedures may include wedge resection or gastrotomy with ligation of the bleeding vessel, but these interventions carry risks related to anesthesia, operative blood loss, and preterm labor. In pregnant patients, the decision to proceed with surgery must be highly individualized and guided by gestational age, maternal hemodynamic stability, fetal condition, and availability of less invasive alternatives. Whenever possible, surgical management should be undertaken in a tertiary-care setting with immediate access to obstetric and neonatal support. Overall, the need for surgical intervention underscores the importance of early recognition and timely endoscopic management of Dieulafoy’s disease to prevent progression to refractory, life-threatening hemorrhage.

## 5. Conclusions

Dieulafoy’s disease represents a rare but potentially serious cause of gastrointestinal bleeding during pregnancy. Most previously reported cases have been characterized by acute and overt hemorrhagic presentation occurring predominantly in the second and third trimesters and requiring urgent endoscopic intervention. Upper gastrointestinal endoscopy remains the diagnostic and therapeutic cornerstone for Dieulafoy’s disease and, when clinically indicated and performed according to established safety recommendations, is safe during pregnancy. Mechanical endoscopic hemostasis is highly effective, associated with low recurrence, and allows continuation of pregnancy to term.

The present report demonstrates that Dieulafoy’s disease may also present in pregnancy as a clinically silent condition, manifested solely by severe and progressive anemia in the absence of overt gastrointestinal bleeding. Pregnancy-related physiological and hormonal adaptations, in fact, may modify the clinical expression of a pre-existing Dieulafoy’s lesion, masking classical symptoms and complicating timely diagnosis. Consequently, gastrointestinal vascular lesions should be considered in pregnant patients with severe, unexplained, or transfusion-dependent anemia, even when gastrointestinal symptoms are absent and non-invasive investigations are inconclusive. Recognition of clinically silent presentation broadens the currently accepted clinical spectrum of Dieulafoy’s disease in pregnancy: increased awareness of this atypical phenotype may facilitate earlier diagnosis, reduce maternal morbidity, and ultimately improve maternal–fetal outcomes.

## Data Availability

Data supporting the findings of this study are contained within the article. No additional datasets were generated or analyzed during the current study.

## Supplementary Materials

The following supporting information can be downloaded at https://www.mdpi.com/article/10.3390/jcm15051894/s1. Table S1: Summary of published cases of Dieulafoy’s disease during pregnancy; Table S2: Endoscopic diagnostic criteria for Dieulafoy’s lesions; Table S3: Differential diagnosis of gastrointestinal causes of anemia in pregnancy; Table S4: Therapeutic options and outcomes reported in the literature; Figure S1: Clinical decision-making flowchart for suspected Dieulafoy’s disease in pregnancy; Images S1–S3: Endoscopic images from the illustrative case report.
