# Supplementary material for: Dieulafoy’s Disease in Pregnancy: Pathophysiology, Clinical Presentation, and Management—A Case Report with Narrative Literature Review
Source: J Clin Med. 2026 Mar 2;15(5):1894. doi: 10.3390/jcm15051894 (PMC12986233; doi:10.3390/jcm15051894)

Table S1. Summary of published cases of Dieulafoy's disease during pregnancy.

Reference	Gestational Age at Presentation	Clinical Presentation	Lesion Location	Diagnostic Modality	Treatment	Maternal Outcome	Fetal Outcome
Si et al., 2017 [5]	Second trimester	Massive upper gastrointestinal bleeding	Gastric lesion	Upper gastrointestinal endoscopy	Endoscopic clipping	Complete recovery	Favorable
Wong et al., 2004 [6]	Second trimester	Acute upper gastrointestinal bleeding with hemodynamic instability	Gastric lesser curvature	Upper gastrointestinal endoscopy	Endoscopic clipping	Favorable	Favorable
Present case	Second trimester	Severe progressive anemia without overt gastrointestinal bleeding	Cardia and gastric body (multiple lesions)	Upper gastrointestinal endoscopy	Endoscopic clipping	Complete recovery	Term delivery

Table S2. Endoscopic diagnostic criteria for Dieulafoy's disease [Refs. 2, 4, 20,21].

Criterion	Endoscopic Description
Active arterial bleeding	Spurting or micropulsatile bleeding originating from a minute mucosal defect
Visible vessel	Protruding submucosal artery through otherwise normal-appearing mucosa, with or without active bleeding
Adherent clot	Densely adherent clot attached to normal surrounding mucosa
Absence of ulcer or mass lesion	No associated ulceration, erosion, or neoplastic lesion

Table S3. Differential diagnosis of upper gastrointestinal bleeding and distinguishing features [Refs. 2, 3, 22].

Condition	Typical Clinical Features	Key Endoscopic Findings
Dieulafoy's lesion	Sudden, massive or recurrent bleeding	Normal surrounding mucosa with visible vessel or adherent clot
Peptic ulcer disease	Epigastric pain, NSAID use	Ulcer crater with inflamed or fibrotic margins
Esophageal varices	Portal hypertension, chronic liver disease	Dilated, tortuous submucosal veins
Mallory–Weiss tear	Bleeding following vomiting or retching	Linear mucosal tear at the gastroesophageal junction
Gastric malignancy (rare)	Weight loss, chronic anemia	Mass lesion or irregular ulceration

Table S4. Therapeutic options for Dieulafoy's disease and considerations in pregnancy [Refs. 3-6, 20, 21,24].

Treatment Modality	Mechanism of Action	Advantages	Pregnancy-Specific Considerations
Hemoclip placement	Mechanical closure of the bleeding vessel	High efficacy, low rebleeding rate	Preferred first-line modality
Endoscopic band ligation	Mechanical strangulation of the vessel	Rapid and effective hemostasis	Safe when anatomically feasible
Epinephrine injection	Local vasoconstriction	Widely available, easy to apply	Adjunctive therapy; use cautiously
Thermal coagulation	Cauterization of the bleeding vessel	Effective when combined with other methods	Avoid deep tissue injury
Angiographic embolization	Selective vascular occlusion	Option for refractory bleeding	Radiation exposure risk
Surgery	Segmental resection of affected area	Definitive treatment	High maternal–fetal risk; last resort

Figure S1. Diagnostic approach to suspected Dieulafoy's disease in pregnancy.

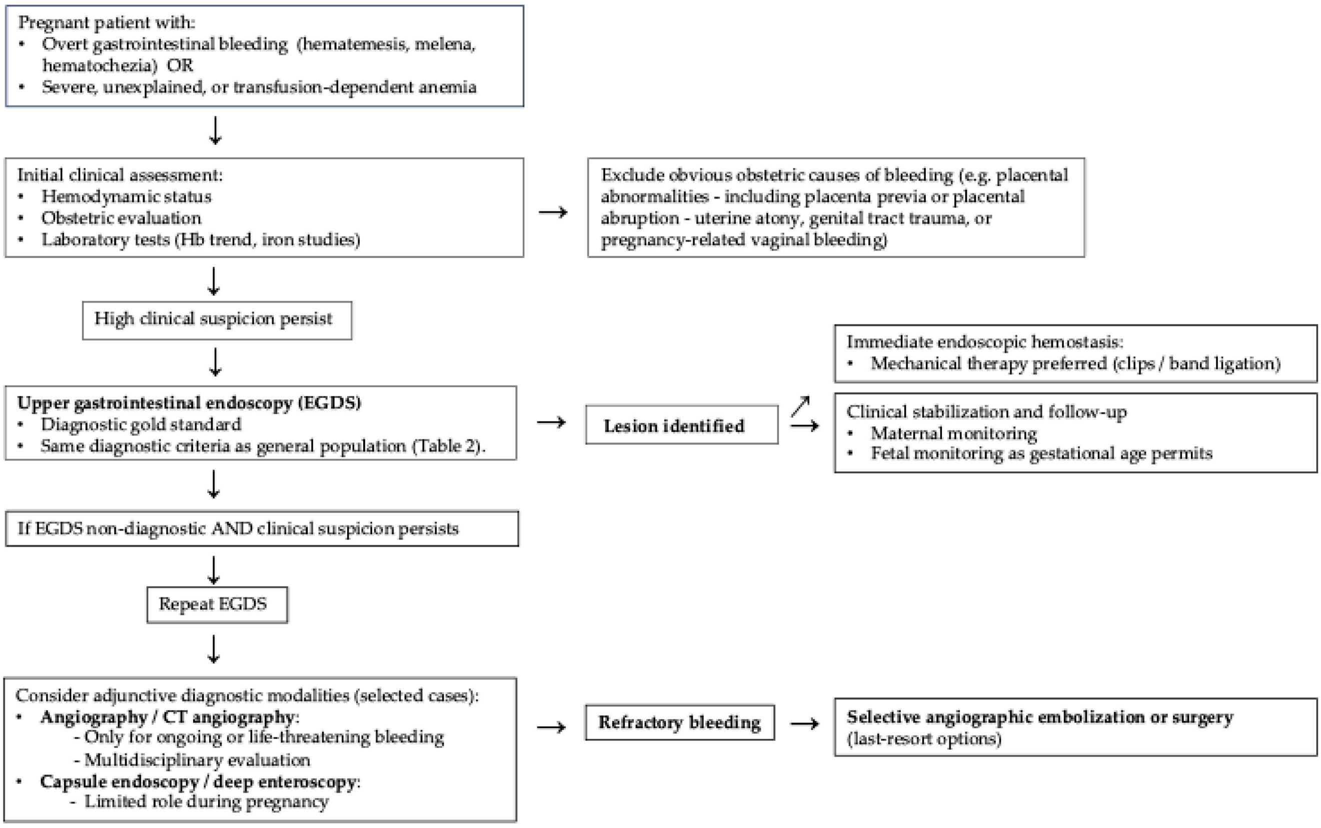

Image S1.

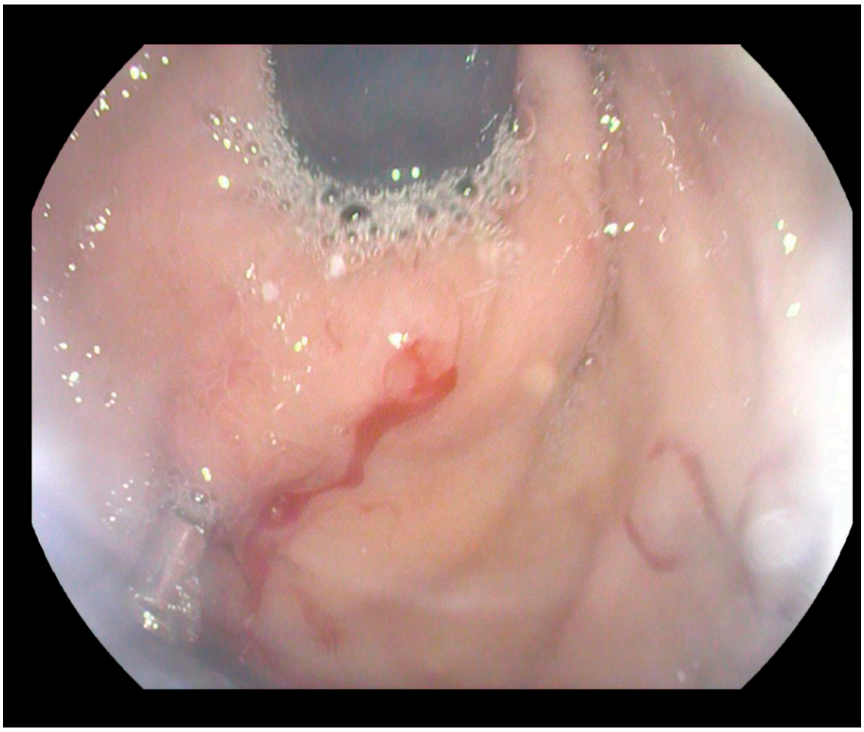

Image S2.

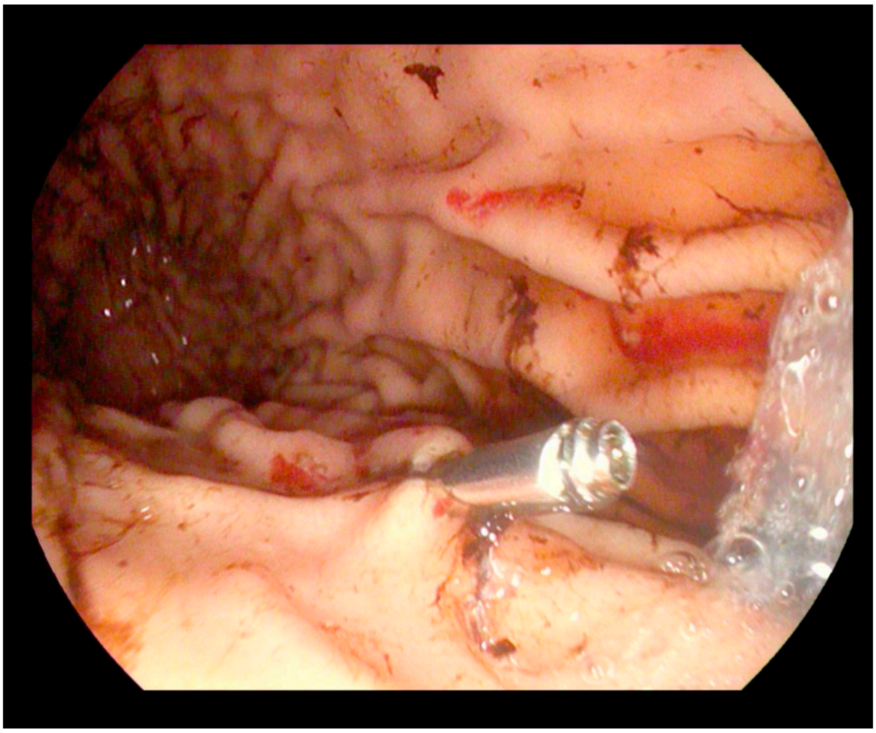

Image S3.

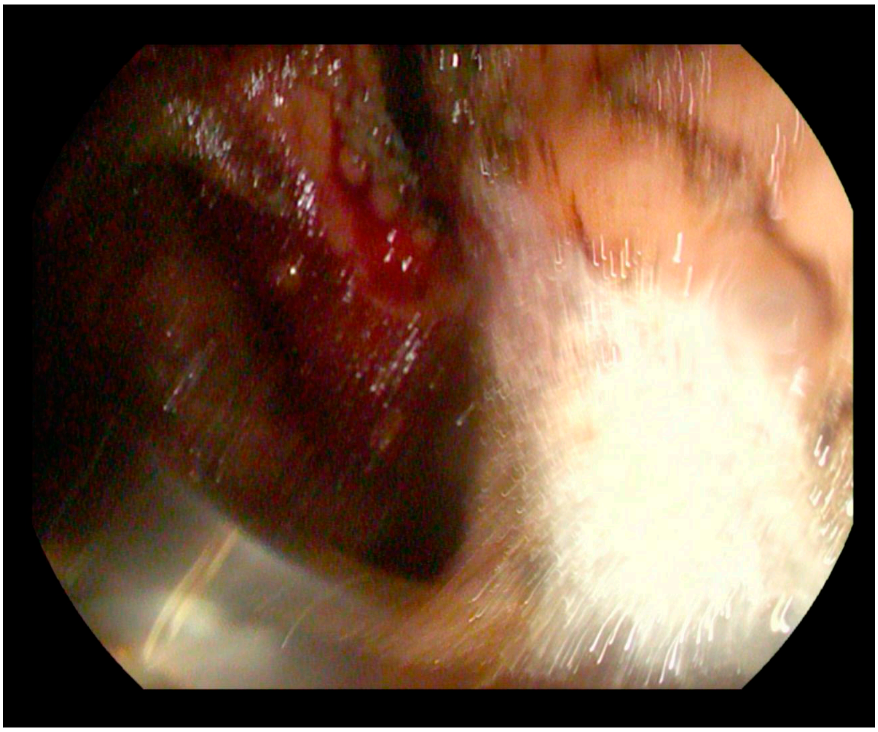

Supplement: Supplementary file 1 [file jcm-15-01894-s001.zip › jcm-4162511-supplementary.pdf]
